# Implementation, efficacy, costs and processes of inpatient equivalent home-treatment in German mental health care (AKtiV): protocol of a mixed-method, participatory, quasi-experimental trial

**DOI:** 10.1186/s12888-021-03163-9

**Published:** 2021-03-30

**Authors:** Johanna Baumgardt, Julian Schwarz, Andreas Bechdolf, Konstantinos Nikolaidis, Martin Heinze, Johannes Hamann, Martin Holzke, Gerhard Längle, Janina Richter, Peter Brieger, Reinhold Kilian, Jürgen Timm, Constance Hirschmeier, Sebastian Von Peter, Stefan Weinmann

**Affiliations:** 1 Department of Psychiatry, Psychotherapy and Psychosomatic Medicine, Vivantes Hospital Am Urban und Vivantes Hospital im Friedrichshain, Charité – Universitätsmedizin Berlin, Vivantes Klinikum Am Urban, Berlin, Germany; 2 Department of Psychiatry and Psychotherapy, Brandenburg Medical School Theodor Fontane, Immanuel Clinic Rüdersdorf, Rüdersdorf, Germany; 3 ORYGEN, National Center of Excellence of Youth Mental Health, University of Melbourne, Melbourne, Australia; 4 Department for Psychiatry and Psychotherapy, University Hospital Cologne, Cologne, Germany; 5 kbo-Isar Amper Klinikum, Region München, Munich, Germany; 6 Department of Psychiatry and Psychotherapy, Technical University of Munich, Munich, Germany; 7 Center for Psychiatry Suedwuerttemberg, Department of Psychiatry I, Ulm University, Ravensburg, Weissenau Germany; 8 Center for Psychiatry Suedwuerttemberg, Zwiefalten, Germany; 9 Gemeinnützige GmbH für Psychiatrie Reutlingen (PP.rt), Academic Hospital of Tuebingen University, Reutlingen, Germany; 10 Department of Psychiatry and Psychotherapy, University Hospital Tuebingen, Department of Medicine of the Tuebingen University, Tuebingen, Germany; 11 Department of Psychiatry II, Ulm University, Günzburg, Germany; 12 University of Bremen, Bremen, Germany; 13 Department for Psychiatry and Psychotherapy, Charité University Hospital Berlin, Berlin, Germany; 14 Psychiatric Hospital and Rehabilitation Unit, Rudolf-Sophien-Stift, Stuttgart, Germany; 15 University Psychiatric Hospital Basel, Basel, Switzerland

**Keywords:** Community mental health care, Crisis resolution teams, Home treatment, Multi-center study, Inpatient-equivalent treatment, Mixed methods, User involvement, Collaborative research, Coproduction

## Abstract

**Background:**

Over the last decades, many high-income countries have successfully implemented assertive outreach mental health services for acute care. Despite evidence that these services entail several benefits for service users, Germany has lagged behind and has been slow in implementing outreach services. In 2018, a new law enabled national mental health care providers to implement team-based crisis intervention services on a regular basis, allowing for different forms of *Inpatient Equivalent Home Treatment* (IEHT). IEHT is similar to the internationally known Home Treatment or Crisis Resolution Teams. It provides acute psychiatric treatment at the user’s home, similar to inpatient hospital treatment in terms of content, flexibility, and complexity.

**Methods/design:**

The presented naturalistic, quasi-experimental cohort study will evaluate IEHT in ten hospitals running IEHT services in different German regions. Within a multi-method research approach, it will evaluate stakeholders’ experiences of care, service use, efficacy, costs, treatment processes and implementation processes of IEHT from different perspectives. Quantitative surveys will be used to recruit 360 service users. Subsequently, 180 service users receiving IEHT will be compared with 180 matched statistical ‘twins’ receiving standard inpatient treatment. Assessments will take place at baseline as well as after 6 and 12 months. The primary outcome is the hospital re-admission rate within 12 months. Secondary outcomes include the combined readmission rate, total number of inpatient hospital days, treatment discontinuation rate, quality of life, psycho-social functioning, job integration, recovery, satisfaction with care, shared decision-making, and treatment costs. Additionally, the study will assess the burden of care and satisfaction with care among relatives or informal caregivers. A collaborative research team made up of researchers with and without lived experience of mental distress will conduct qualitative investigations with service users, caregivers and IEHT staff teams to explore critical ingredients and interactions between implementation processes, treatment processes, and outcomes from a stakeholder perspective.

**Discussion:**

By integrating outcome, process and implementation research as well as different stakeholder perspectives and experiences in one study, this trial captures the various facets of IEHT as a special form of home treatment. Therefore, it allows for an adequate, comprehensive evaluation on different levels of this complex intervention.

**Trial registration:**

Trial registrations: 1) German Clinical Trials Register (DRKS), DRKS000224769. Registered December 3rd 2020, https://www.drks.de/drks_web/setLocale_EN.do; 2) ClinicalTrials.gov, Identifier: NCT0474550. Registered February 9th 2021.

## Background

Research showed that assertive community treatment (ACT) and home treatment (HT) is often preferred over inpatient treatment [1, 2]. For instance, acute inpatient treatment can be perceived as having a stigmatizing effect [3] and can therefore sometimes be rejected or delayed by service users. This may negatively affect the course of illness and may lead to longer recovery times. Furthermore, psychiatric inpatient treatment can be associated with prolonged phases of absence from home, which may impair social participation and may increase overall social costs for service users [4]. Additionally, people who have to look after children or relatives may sometimes not able to leave for inpatient treatment. Therefore, in many countries, assertive outreach services have also been implemented for acute mental health care [5]. Since comparative studies showed positive effects of these services on several outcomes, they are recommended by international and national guidelines [6–8].

In Germany, the fragmentation of the mental health care system has long impeded such needs-orientated, comprehensive and coordinated care for people experiencing mental health crises. Efforts had been made to overcome cross-sectoral boundaries and to promote integrative, flexible, outreach models of mental health care in Germany [9, 10]. Due to legal conditions, these models could not be implemented nationwide and remained temporarily limited to specific catchment areas, health insurances or specific diseases [11, 12]. In this context, outreach care models such as HT or ACT were mostly implemented as pilot projects. Intense mental health care for acute psychiatric crises was almost exclusively provided in inpatient hospital settings. There were only a few incentives to establish multi-professional outreach teams to prevent hospitalizations.

To overcome these shortcomings, in 2018, the legal paragraph 115d was introduced into the German Social Code, Book Five (SGB-V). This paragraph enables psychiatric departments and hospitals in Germany to deliver “Inpatient Equivalent Home Treatment” (IEHT) (“stationsäquivalente Behandlung”). This internationally well-known construct allows for standard outreach treatment for the first time in Germany as a replacement (“equivalent”) for inpatient treatment [13]. Thus, IEHT is acute psychiatric treatment with a similar intensity and flexibility to inpatient treatment, but delivered in the users’ home by mobile, multi-professional teams, including a psychiatrist [14]. Key ingredients are daily home visits, medical rounds by mental health specialists, regular multi-professional team meetings and a round-the-clock availability of the team or the hospital [15].

German expert opinions and exploratory assessments conservatively assume that 10–15% of all service users treated in an inpatient setting in Germany would be suited for IEHT. Taking into account recent hospital statistics, this would translate into to approximately 100,000–150,000 service users per year [16]. By October 2020, about 50 hospitals had implemented IEHT [17–20], with the trend predicted to increase: Up to 650 psychiatric departments are expected to gradually include forms of IEHT into their range of treatment options over the next years. Against this background, evidence on the effectiveness and implementation models of IEHT are of substantial relevance for the further development of the mental health care system in Germany.

Given the short time period, there is still a lack of elaborated implementation guidelines, fidelity scales or detailed evidence on IEHT. In this context, international guidelines and evidence are of only limited use, for several reasons: Firstly, IEHT is a unique construct within the German healthcare system and fulfills only some of the internationally defined criteria for HT or Crisis Intervention Treatment. It differs, for instance, from HT in Great Britain as it is less flexible and requires at least one personal contact with users per day, cannot be gradually phased out and is associated with strict criteria for reimbursement. Secondly, it cannot be compared with ACT as these services have been designed for long-term support, in contrast to the limited scope of IEHT, which is restricted to times of acute crisis [21, 22]. In addition, the transferability of results from international studies is limited since complex interventions such as ACT are context-dependent and effectiveness may vary according to institutional frameworks as well as professional, societal and economic incentives [23–25]. For these reasons, a mixed-method, quasi-experimental trial of IEHT was started in 2020, named AktiV (German: “**A**ufsuchende **K**risenbehandlung mit **t**eambasierter und **i**ntegrierter **V**ersorgung: Evaluation der stationsäquivalenten psychiatrischen Behandlung (StäB nach §115d SGB V)”; English: “Outreach Crisis Intervention with a team-based and integrative model of treatment (AKtiV Study): Evaluation of the Inpatient Equivalent Home Treatment (IEHT according to the German Social Code Book §115d SGB V)”.

## Methods/design

### Aim, design and setting of the study

The overarching goal of the AKtiV trial is to examine implementation processes, treatment processes, clinical efficacy, costs, and subjective experiences of IEHT compared to inpatient treatment from the perspective of service users, relatives or informal caregivers, staff and other stakeholders in mental health care. To maximize the transferability of study results and to cover a broad spectrum of IEHT experience, 10 hospitals from different regions in Germany (e.g. rural, urban, east, west) are participating in this study. Combining routine data, primary data and prospective follow-up data, the study results will be compiled in a comprehensive database. Furthermore, the combination of clinical and health economic data will enable the assessment of costs and benefits from a national perspective, an important characteristic, given that there are only a few studies with health economic evidence dealing with acute outreach mental health care [6, 26–29]. The qualitative evaluation of IEHT processes and outcomes uses a collaborative-participatory approach that aligns with current demands for more user orientation and/or the involvement of people and researchers with relevant lived experience in the process of developing interventions and their evaluation [30]. The trial’s mixed-method design corresponds with current standards of empirical social research enabling the triangulation of hypothesis-confirming quantifiable factors and hypothesis-generating qualitative aspects [31]. By parallelizing quantitative and qualitative data on the one hand and routine data on the other with primary data, data on implementation processes and data on treatment processes, different facets from different perspectives and levels of IEHT are targeted. This allows for a comprehensive, holistic assessment of this innovative treatment option.

### Characteristics of participants

The study population consists of service users seeking IEHT and their caregivers living in the same household, as well as staff delivering IEHT and other stakeholders in the mental health care system and politics.

#### Service Users

- The recruitment of the intervention group (IG) starts with users seeking IEHT offered by the participating study sites. Admission to IEHT takes place via the usual referral pathways. When the prospective participant reports to the hospital, staff will check whether the person fulfills the official IEHT criteria required:
  An acute mental health crisis that requires inpatient treatment
  Social and living conditions that allow for home visits and private conversations
  Informed consent of all adults living in the service user’s place of residence
  In the case of children living in the user’s household, there should be no associated child welfare risk [14].

If the person fulfills all IEHT criteria, he or she will be informed about the opportunity to receive IEHT as an alternative to regular psychiatric inpatient treatment. If the service user agrees to receive this form of treatment, the study staff will check if he or she fulfills the inclusion criteria of the AKtiV trial:

- No acute suicidality or aggressiveness towards others requiring hospital admission
- Main diagnosis within the ICD codes F0X, F1X, F2X, F3X, F4X, F5X, or F6X
- Permanent residence in the catchment area of the hospital delivering IEHT
- Not being subject to any form of commitment order
- Ability to provide informed consent
- No participation in an interventional study
- Sufficient German language skills
- Absence of substantial cognitive deficits as indicated by severe organic brain disease
- No diagnosis of intellectual impairment
- Admission no longer than 7 days ago

If these criteria are fulfilled, study staff will present the study design of the AKtiV trial to the service user and ask for participation. In the case of consent, study staff will request the person’s signature to confirm receipt of study information and their willingness to participate in the study.

b) The control group (CG) comprises service users receiving treatment as usual (TAU), i.e. regular inpatient psychiatric treatment according to the hospital standards and must also fulfill the inclusion criteria named above. Propensity Score (PS) Matching will be carried out to find the best matching partner to the IG.

#### Relatives/informal caregivers

One close relative or informal caregiver living in the same household of each service user from the IG as well as from the CG will be informed and their consent requested for participation in the study.

#### Staff

All members of the IEHT teams at all study sites, including team leaders, will be asked to participate in a both quantitative and qualitative assessment of job satisfaction, stress levels and treatment processes (forms of therapy, location, point of time during treatment, collaboration with outpatient stakeholders, etc.).

#### Other stakeholders

In addition to the IEHT team and hospital staff, this study includes stakeholders from community psychiatry (such as outpatient consultant psychiatrists, communal psychiatric nursing, social participation and rehabilitation units) as well as experts for mental health policy, practice and research as study participants.

### Intervention

After admission to IEHT, staff will conduct an individual needs assessment based on which the team will develop a treatment plan. This plan may include treatment goals, various measures such as medication, psychotherapy, training and other daily or therapeutic activities, as well as therapeutic interactions with relatives, informal caregivers, legal guardians, and other persons from the participant’s social network. Furthermore, it will consider potential triggers for future crises, list former treatments, and individual preferences. Therapeutic interventions are adapted to the user’s needs daily. The team establishes daily contacts that can either take place at home, at the hospital, or at any place the service user feels comfortable with, but six encounters per week with service users must be realized outside of the hospital. In the weekly consultation by the psychiatrist in charge, the treatment progress will be reflected upon, and further interventions will be planned, such as therapeutic sessions or a change of medication. Treatment is realized according to the available resources and standards of the study sites. Service users will be discussed extensively in regular, inter-professional IEHT team meetings at least once a week, involving medical staff and nurses with participation of at least one psychologist, social worker or member of another professional group. Discharge planning follows inpatient procedures.

### Outcomes and hypotheses

**Fig. 1:**
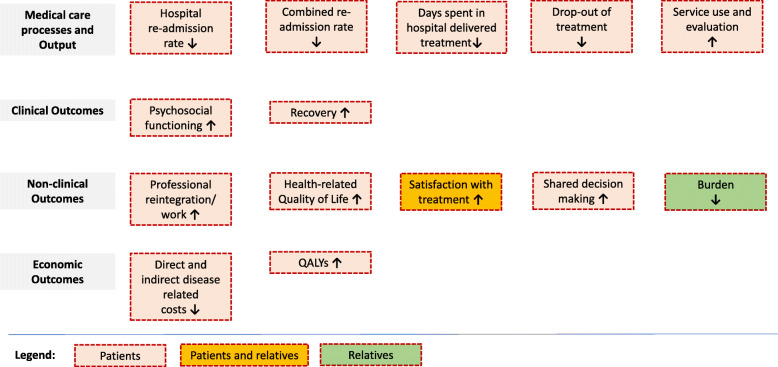
Main quantitative outcomes and hypotheses of the AKtiV trial

The main quantitative outcomes and hypotheses of the trial are shown in Fig. 1. The hospital re-admission rate within 12 months of the index crisis that originally led to the need for immediate admission either to IEHT or inpatient treatment serves as the primary outcome. This outcome has been used in most international home treatment studies [32]. The re-admission rate is by no means a perfect quality indicator of psychiatric care [33]. Nevertheless, it is an indicator of successful acute treatment, recovery and needs met within community mental health care [34].

### Module-based trial

Clinical and research staff from six institutions from different regions in Germany collaborate in the AKtiV trial which is divided into five modules: Module A examines quantitative outcomes among service users and relatives or informal caregivers; Module B uses a qualitative and collaborative-participatory methodology to map the care providers’ experiences; Module C analyzes the implementation processes and the treatment processes of IEHT; Module D evaluates health care costs, and Module E is in charge of all biometric and statistical questions relating to the trial.

### Recruiting sites

Study sites were selected ahead of the trial in order to avoid disturbances during the recruiting period. Ten IEHT teams, associated with ten different psychiatric hospitals, from both urban and rural areas, located in different regions of Germany, agreed to participate in the trial: The Immanuel Clinic Rüdersdorf, Vivantes Hospital Am Urban (Berlin), Vivantes Hospital Neukölln (Berlin), and Charité – University Medicine Berlin in the North East, and the hospitals ZfP Zwiefalten, Ravensburg, Reutlingen, Isar-Amper Hospital Munich, University Clinic Tübingen and the Hospital ZfP Reichenau, in the South West of Germany. IEHT implementation had been started in these study sites between July 2018 and August 2020. The heterogeneity of hospitals is a strength of this trial as it allows for IEHT to be analyzed in different care settings and different arrangements.

#### Module A – quantitative evaluation among service users and relatives or informal caregivers

Module A will investigate whether IEHT is superior in comparison to TAU regarding the outcomes outlined in Table 1. Assessment tools and points are presented ibid.

**Table 1:** Outcomes and assessments among service users and relatives or informal caregivers

Cohort	Outcome	Assessment tool	Study point		
			Baseline	6-Months-Folllow-Up	12-Months-Follow-Up
**Service user**	**Re-admission rates**	German version of the Client Sociodemographic and Service Receipt Inventory (CSSRI-D) [35]	x^**a**^	x	x
	**Continuity of care**	CSSRI-D	x^**a**^	x	x
	**Health-related quality of life**	German version of the EuroQoL Five-Dimensional Five-Level Questionnaire (EQ-5D-5L) [36]	x^**a**^	x	x
	**Psycho-social functioning**	German version of the Health of the Nation Outcome Scales (HoNOS) [37] and the Personal and Social Performance Scale (PSP) [38]	x^**a**^	x	x
	**Work**	CSSRI-D	x^**a**^	x	x
	**Recovery**	German version of the Recovery Assessment Scale (RAS-G) [39]	x^**a**^	x	x
	**Service use and evaluation**	CSSRI-D	x^**a**^	x	x
	**Satisfaction with treatment**	Self-developed by authors	x^**b**^		
	**Shared decision making**	German version of the 9-item Shared Decision Making Questionnaire (SDM-Q-9) [40]	x^**a**^	x	x
**Relative or informal caregiver**	**Burden**	German version of the Involvement Evaluation Questionnaire (IEQ-EU) [41]	x^**a**^		
	**Satisfaction with treatment**	Self-developed by authors	x^**b**^		

^a^ assessment at the beginning of index treatment period (up to 7 days after admission); ^b^ assessment at the end of treatment period (up to 7 days before or after dismissal of treatment), = data regarding these outcomes will be analyzed further by Module C

### Power calculations

Power calculations for the primary outcome were based on international RCTs included in the Cochrane Review [7] as well as on an analysis of routine data on IEHT from the participating study sites. In a first step, weighted mean re-admission rates by Hoult et al. [42], Fenton et al. [43], and Johnson et al. [44] as presented in the Cochrane Review were used. This led to an expected ratio of 70% re-admissions in the CG versus 45% in the IG within 12 months after admission, or rather a quotient of re-admission rates between IG and CG of approximately 1/3 (exact 0.358). However, data from nine participating study sites from overall *N* = 37,007 service users receiving either IEHT or inpatient treatment within 12 months showed re-admission rates among inpatient treated service users of 52.1%. This is about 20% lower than the internationally reported numbers. Analysis in a small pilot study (*n* = 86) site showed an even smaller difference between the re-admission rate of IEHT (37.2%) and inpatient treatment (46.5%) in a 12-month-follow-up-assessment [45], i.e. a reduction quotient of about 1/5. Following this data, expecting a quotient of readmission rates between IEHT and TAU of about 1/3 as internationally observed seemed to be too optimistic. Thus, for the trial presented, we used our own TAU value of 52.1% and combined all data sources in order to receive a realistic IEHT rate by a weighted mean of the reduction factors applying a 10% higher weight for the international value than for the small pilot study factor. As a result, an IEHT rate reduction by a quotient of about 0.288 was obtained. Thus, an admission rate of 37.4% after IEHT is expected and should be detected. A power calculation using nQuery Advisor 7.0. with an alpha of 5% and a power of 80% revealed in a two-sided chi square test a total number of 360 service users (n_IG_ = 180, n_CG_ = 180) that must be included into the trial to show the effects named above. Assuming a non-response rate of 10% as shown in two pilot studies [46, 47], approximately 400 service users (n_IG_ = 200, n_CG_ = 200) have to be canvassed in order to achieve the optimum number of participants for the trial.

### Recruitment

Recruiting will take place consecutively in the participating study sites. Trained scientific study staff will recruit service users for the IG and the CG as described above and will be monitored regularly. Study staff will conduct screening as well as information about the study, inclusion into the study, and study assessments, but will not be involved in care delivery. Since randomization of study participants was judged to be both ethically and logistically infeasible (**see** the “Limitations” section), PS Matching with regard to age, gender, main psychiatric diagnosis, and number of previous stays in the corresponding hospital within 2 years, is used in this trial to generate a CG. Matching pairs will be identified through a special PS function that has been developed specifically for the AKtiV study (further described in the “Module E” section). An inpatient treatment service user will be asked to participate in the CG if the difference in his or her PS is < 0.1.

The recruitment period of the AKtiV study lasts 12 months. The number of IEHT cases in the preceding 12 months regarding the preparation of this manuscript ranged between 44 and 209 with a mean of approximately 120 IEHT cases per study site. Available treatment units at participating study sites currently range from five to 21 resulting in 130 IEHT units overall (mean = 13 IEHT units per study site). The mean stay of IEHT service users during the index period in the preceding 12 months was approximately 32 days. Beyond this background, the inclusion of 180 IEHT users into the trial within 12 months across all ten study sites is ensured. Recruitment of the same number of participants for CG is unproblematic in all the study sites since the amount of inpatient treatment units outnumbers the amount of IEHT in all hospitals by far.

### Assessments

All assessments are conducted face to face either in the hospital, at the service user’s home or in his or her social surroundings. The sequence of assessments in each study cohort is outlined in Table 1. The following incentives for study participation are provided: service users receive 50 € after completing the 12-month-follow-up assessment; relatives or informal care givers of service users receive 20 € after completing the baseline assessment.

#### Module B – qualitative evaluation among care stakeholders

##### Collaborative-participatory evaluation of the service users’ and caregivers’ experiences

This module exclusively uses hypothesis-generating qualitative methods to explore experiences with IEHT from a multi-stakeholder perspective. The following research questions are examined in detail: 1. How do users and caregivers experience IEHT? 2. Which characteristics of IEHT (interaction and communication, staff attitudes, aspects of the delivered services, etc.) are considered helpful or impedimental? 3. What are the specific components of good outreach care as defined by service users? 4. What are the modes of action and confounding factors of IEHT? To answer these questions a participatory approach will be used, involving researchers *with* and *without* lived experience of mental distress during the whole research process [30]. This is to ensure that the focus of knowledge production is not primarily guided by a clinical but rather by a service user perspective [30]. In order to acquire an immediate view of everyday treatment practices in IEHT, participant observation will be used as the main qualitative research tool in this trial [48]. The potential of this technique is to provide access to some of the reflective and overarching aspects of lived experience in situ. In the present study, tandem teams of researchers with and without lived experience will carry out the observation before, during and after each contact with an IEHT team for several days. After having completed the index treatment episode, semi-structured follow-up interviews with the users, caregivers and involved staff will be conducted by the tandems, exploring experiences and evaluations of IEHT, and looking back on the past treatment episode. Qualitative data collection and analysis will be conducted in an iterative process according to Grounded Theory Methodology [49].

In a second step, specific components of “good outreach crisis support” will be developed (according to IEHT) from a service user’s perspective, based on the existing evidence and the collected empirical data. These components should be further developed to a) a questionnaire recording “Patient Reported Experience Measures” (PREMS) with IEHT [50] and b) a logical diagram depicting the mechanisms of change of this complex intervention, following the UK Medical Research Council’s guidelines [51].

##### Evaluation of the IEHT’s potentials to promote cross-sectoral care for severe mental illness (SMI)

Both IEHT and the underlying legal framework were primarily introduced to strengthen cross-sectoral care and thus collaboration and coordination between psychiatric hospitals with other stakeholders involved in treatment and support of people with SMI. This sub-project examines the status quo of intersectoral linkages between IEHT teams and community psychiatric social and rehabilitation services. To this end, the intensity and quality of cross-sectoral cooperation should be measured using Social Network Analysis [52] in catchment areas of two study regions. Similar to Nicaise et al. the intensity (contact frequency, resource sharing) and quality of cross-sectoral collaboration [53] is operationalized and recorded for almost all services and institutions in two catchment areas. Finally, ties between the stakeholders should be visualized to identify brokerage roles but also gaps within the care networks. These findings will be framed and contextualized by a qualitative analysis of key stakeholders’ experiences with intersectoral collaboration in the selected catchment areas. For this purpose, expert interviews are conducted and evaluated using qualitative content analysis [54]. The results of the described evaluation should be discussed and validated within a group of experts for mental policy, practice, and research.

#### Module C – routine data analysis and process evaluation

##### Evaluation of implementation processes and treatment processes

Apart from a few, hardly transferable recommendations on the implementation of HT from international guidelines [1], the specific requirements are defined in the form of broad regulatory conditions in policy documents and standards for performance documentation by the German Federal Institute for Drugs and Medical services (BfArM). This has led to variations in the implementation of IEHT depending on to structures and treatment processes. Therefore, Module C examines this complex topic on several levels in two separate module parts – Module C 1 and Module C2. Module C1 examines the structures, configuration, organization and services provided by the IEHT teams of the included study hospitals and compares them with existing internationally evaluated HT concepts. Module C2 aims at triangulating both quantitative and qualitative data from the outcome evaluation of the Modules A and B to deduct causal mechanisms about how the implementation of specific components of IEHT affects downstream impacts for service users, caregivers and employees. Finally, a routine data set is to be developed that enables a continuous cross-clinic evaluation of the structures and processes of IEHT and the assignment to different IEHT models.

### Method

Module C1 includes a) institutional and structural data from the study departments, b) routine data about service provision, c) additional data about IEHT implementation, and d) quantitative and qualitative primary data collected from IEHT team members about their work satisfaction. To explore treatment processes, personal routine data according to § 301 SGB V will be used. This includes performance data involving admission, working and discharge diagnoses, information about therapy times, pretreatment, and transfer from IEHT to inpatient setting or vice versa. Routine data will be complemented by additional personal user data about the psycho-social background, treatment course, and illness chronicity. Based on qualitative surveys with the IEHT team members, comprehensive information about the implementation process will be collected. In addition, the employee’s job satisfaction will be assessed using quantitative measures.

A comparison between the type of services provided by different professional groups and the amount of time dedicated to service users, therapeutic groups as well as organization and administration, IEHT team structure and data about service users enables an exploration of the impact of the IEHT team processes on treatment output and outcome.

Furthermore, Module C analyses quantitative data assessed by Module A regarding satisfaction with the provided services as well as aspects of shared decision making from the perspective of users and their relatives or informal caregivers. In addition, these analyses will be related to quantitative data regarding employees’ satisfaction and data from qualitative focus groups that will be carried out in teams providing IEHT. The latter make it possible to consider primarily non-quantifiable data on the relevant effects of team building, cohesion and the thematic foci of IEHT, which in turn determine the way in which IEHT services are provided and structured.

#### Evaluation of different pathways to IEHT

Basically, there are two different ways for service users to enter IEHT: a) Direct admission, i.e. totally replacing an inpatient stay or b) admission from a psychiatric inpatient unit, i.e. following and shortening an inpatient stay. So far, no examination has taken place of the factors responsible for these different access pathways, whether outcomes of these pathways differ from each other. The key idea of Module C2 is that there are distinctly different indications for direct admission vs. admission following an inpatient stay. For instance, service users caring for children or relatives may prefer IEHT upfront, while domestic conflicts may be a reason for entering IEHT after an episode of inpatient treatment.

Module C2 aims at studying three hypotheses:

1. Users accessing IEHT directly differ significantly from users who receive inpatient treatment in advance in terms of disease severity, course of treatment and treatment satisfaction. If patients are directly admitted to IEHT the effectiveness regarding clinical improvement and re-admission rate is particularly high.
2. Admission pathways and outcomes can be explained by various factors: If admissions are managed by a central admission unit, the number of direct IEHT treatment episodes is higher compared to other access ways (e.g. via inpatient units, outpatient departments or emergency departments). Other factors include the domestic situation, symptom severity, self-risk or risk for others as well as substance abuse and somatic comorbidity.
3. There are differential indications, which suggest a direct admission into IEHT or where an IEHT episode makes more sense after an initial phase of inpatient treatment.

### Method

To address these research questions, service users’ characteristics (such as age, gender, diagnoses, symptom severity, psycho-social functioning, social network, marital status, socioeconomic status, and previous treatment episodes), treatment processes and outcomes regarding the two admission pathways will be examined. A central goal of Module C2 is to gain knowledge about how to improve indication of IEHT and as well as process and outcome quality.

We will use both single- and multi-center analyses. The multi-center study will include user-specific admission data, data about the treatment process as well as outcome data across all recruiting sites. The single-center analysis will include more detailed quantitative data (such as patients’ aggression etc. at direct IEHT admission vs. inpatient treatment admission) as well as qualitative data, which will not be available in all study centers. It will be conducted at the Munich study site, currently holding the largest German IEHT unit with 20 treatment places.

Module C2 will comprise three work packages. In work package 1 potential influencing factors for the two access paths outlined above will retrospectively be identified from medical records of 100 consecutive service users of the Munich study department. Group comparisons will help to identify a set of variables which can predict direct IEHT admission vs. admission after an inpatient stay. In work package 2, a core set of predicting variables will be generated, which will be prospectively collected in all study departments. These data will then allow for a multi-center analysis regarding the prediction of access routes. Moreover, the multi-center analysis will cover comparisons of treatment outcomes of patients having entered the two access routes. In work package 3, an additional mixed-method single-center analysis regarding different entry pathways to IEHT will be performed. Regarding quantitative data, the more detailed variable set identified in work package 1 will be used to prospectively collect relevant data in the Munich study center (approx. *n* = 140). This quantitative analysis will be extended by qualitative data collection conducted in Munich addressing the different access routes to IET from a service user, caregiver and employee perspective.

#### Module D – health economic evaluation

There are only a few health economic evaluation studies associated with acute mental health home treatment/ crisis intervention treatment compared with treatment as usual [28, 55]. Two studies from the UK show a reduction of costs for mental health care as a consequence of reduced inpatient stays [27, 56]. The only German study found that despite a longer duration of treatment, acute home-based mental health care according to the Ulm/Guenzburg model [57] is less expensive compared to inpatient treatment [26]. Due to conceptual differences between the models and methodological limitations, these results cannot be directly transferred to IEHT. Therefore, Module D compares health care costs of IEHT and TAU from a societal perspective (direct and indirect costs) [29].

### Method

Following the net benefit approach, a cost-utility analysis (CUA) based on primary data will be conducted [58–60]. The aim of this analysis is an estimation of the maximum willingness to pay (MWTP) needed for gaining one quality-adjusted life year (QALY) through IEHT compared with TAU. The CUA from a societal perspective is based on a complete analysis of direct and indirect health costs as well as measuring subjective quality of life with a preference based method [61, 62]. Registering all health care costs is only possible by directly asking service users. This is because mental health care services in Germany are funded by various different bodies according to the Social Code Book and other laws [60, 63]. Therefore, direct and indirect medical and mental health care costs will be assessed with the German Version of the Client Socio-Demographic Service Receipt Inventory (CSSRI-D) [35, 64]. Health care related costs will be calculated by multiplying units of care with relevant, published unit costs values [60, 65, 66].

Quality adjusted life years (QALYs) will be estimated on the basis of health states estimated by means of the EQ. 5D-5L [67, 68] and utility values from the German population provided by Ludwig et al. [69].

Based on the “state of the art” discussion regarding the choice of threshold values for the MWTP, threshold values of 25,000, 50,000, 75,000, and 100,000 € will be taken as the basis for net benefit regression models [70]. However, thresholds can always be differentiated further.

#### Module E – biometry and data management

Module E relates to the creation of a data protection concept, a study database, and a data management plan for all modules of the trial. In line with the data protection concept, the data management plan will contain technical details of assessment, transfer, storage and provision of data. Furthermore, we will deal with quantitative data analysis.

### Matching

Before starting the recruitment process, logistic regression analyses of the previous year’s data from all participating study sites were conducted to calculate PS functions. Data included those of service users of IEHT as well as inpatients fulfilling the trial’s inclusion criteria over a period of 12 months. “Participation in IEHT” (yes/no) was chosen as a dependent dichotomous variable, along with the independent variables age, sex, primary diagnosis (FX), and number of psychiatric hospitalizations for the last 2 years in the corresponding study site. These analyses provided every study site with a specific function for calculating their PS given their respective regression variables. By means of this function, each participant included into the IG will be matched with the most comparable service user treated in inpatient units of the participating study centers.

### Handling of dropouts and missing values

Intention-to-treat (ITT) analysis will be conducted to avoid selective reporting and biases in the analysis due to possible non-random attrition of participants and missing values [71]. Additionally, multiple imputation will be conducted for missing values.

### Data analysis

Regression variables used for matching as well as propensity scores will be tested for homogeneity in an exploratory manner. Descriptive statistics will be performed for all baseline values and outcomes at follow-up. Differences between intervention and control group will be analyzed based on the endpoint or working hypothesis categories “primary”, “secondary”, and “tertiary” following the null hypothesis “No difference between intervention and control group.” Among service users, these categories applied as followed: P = hospital re-admission rate; S1 = combined re-admission rate (inpatient + partly inpatient + IEHT), S2 = inpatient hospital days; S3 = continuity of care/withdrawal from treatment, S4 = health related quality of life, S5 = psycho-social functioning, S6 = job integration, S7 = recovery, S8 = satisfaction with care, S9 = shared decision making; impact of T1 = diagnosis groups, T2 = region, T3 = employment status, T4 = chronicity of disorder, T5 = additional therapies. Assessments among relatives or informal caregivers will be analyzed by Module C and belong to secondary endpoints: S10 = burden, S11 = satisfaction with care. Thereby, P1, S1, S2, S4, S5, S6, and S7 refer to the time span of 12 months (main analysis) and 6 months (supporting analysis) after baseline assessment. S3, S8, S9, S10 and S11 refer to the index episode.

All statistical analyses will be conducted with SAS. Prior to the end of data assessment, a statistical analysis plan (SAP) will be created determining statistical methods, e.g. for transformations, imputations and, analyses of quantitative data to be processed especially for Module A and partly Module C. A synthesis of results from the different Modules will be described procedurally in the SAP. The main features of the plan are as follows:

- Descriptive analyses will be conducted for all quantitative data. For all categorical variables, numerical and percentage data will be calculated separately for the IG and for the CG. For metric variables, mean, standard deviation, median, minimum, and maximum will be calculated additionally. We will test whether balance between intervention and control was achieved using logistical regression and the PS.
- The primary outcome will be tested deductively using a two-sided Chi-square test with a significance level of α = 5% and the null hypothesis “equal rates of readmission for IEHT and TAU”.
- Secondary outcomes will be tested in an explorative manner with α = 5%. Assumption for standard distribution will be tested with the Shapiro-Wilk test [72]. If data is normally distributed, paired t-test will be applied. If data is not normally distributed, the Mann-Whitney test will be used. Baseline correction will be performed by taking the individual baseline value as factor in a sensitivity logistic regression.
- Tertiary outcomes will be tested in an explorative manner using an overall logistic regression with “re-admission” (yes/no) as dependent dichotomous variable and diagnosis, urban-rural, workplace and chronicity of disease as well as other variables showing significant influences as independent variables.

Results will be interpreted regarding reference values and similar study populations.

## Discussion

“AKtiV” is the first quasi-experimental multi-center study using a pre/post assessment evaluating IEHT. The results will add to the knowledge of clinical as well as subjective and health economic effects of HT concepts similar to IEHT. This may directly transform into clinical practice since specific conclusions concerning the implementation of IEHT in the German mental health care system can be drawn from the study’s results. The study collects information on how psychiatric departments may establish more user-centered and needs-based forms of crisis support at home, and how informal caregivers may be included during these processes in a meaningful way. Different admission pathways to IEHT will be analyzed, resulting in prototypes and participant clusters and enabling the comparison of different forms of team organizations (e.g. ward-integrated vs. independent teams) that will help to further implement IEHT in Germany and HT in general. The collaborative-participatory approach of the study aims at assuring that our results are not only relevant to clinical stakeholders but also considers the research priorities of service users and caregivers. Since the feasibility of IEHT has not been proven, the results of the AKtiV study are important for health policy and various stakeholders to further develop this specific HT model and the underlying legal framework.

### Limitations

One limitation of the “AKtiV” study is the lack of direct randomization of study participants. Randomization is not possible as IEHT already is standard care and a preference-based alternative to psychiatric inpatient treatment. Furthermore, the randomization of service users in the CG may lead to conflicts if they have important reasons to prefer IEHT. This could e.g. be the case if a user is a single parent care giver or is caring for an elderly person living in the same household. On the other hand, some service users may not or cannot be treated at home even though they fulfill the inclusion criteria. Moreover, the fact that the number of IEHT units per clinic ranges from 5 to 21 makes randomization challenging. In addition, samples from cohort studies appear to be more representative for clinical routine samples than samples derived from RCTs because they are not biased. Against this background, it was decided to carry out a quasi-experimental cohort study with PS matching, rather than a randomized controlled trial. Another limitation is the relatively short follow-up period. Since funding guidelines only allow for a study period of a maximum of 3 years, the final follow-up-assessment cannot be longer than 12 months. Even though a longer observation period would be desirable in order to analyze the long-term effects and sustainability of IEHT, the current trial duration is sufficient for an initial methodologically sound evaluation of this new home treatment model.

## Data Availability

Study material and data will be available upon request.

## Abbreviations

IEHT: Inpatient Equivalent Home Treatment
ACT: Assertive community treatment
HT: Home treatment
AKtiV: Outreach Crisis Intervention with a team-based and integrative model of treatment (Aufsuchende Krisenbehandlung mit teambasierter und integrierter Versorgung)
IG: Intervention group
CG: Control group
TAU: Treatment as usual
PS: Propensity Score
PREMS: Patient Reported Experience Measures
SMI: Severe mental illness
BfArM: German Federal Institute for Drugs and Medical services
CUA: Cost-utility analysis
MWTP: Maximum willingness to pay
QALY: Quality-adjusted life year
CSSRI-D: Client Socio-Demographic Service Receipt Inventory
ITT: Intention-to-treat
SAP: Statistical analysis plan
MHB: Brandenburg Medical School Theodor Fontane
GCP: Good Clinical Practice
DFG: Good scientific Practice
MRC: Guideline of the Medical Research Council
SOP: Standard Operating Procedures
KKSB: Competence Center for Clinical Trials
GDPR: General Data Protection Regulation
BDSG: German Federal Data Protection Act
SGB: Social Security Law Book
GBA: Federal Joint Committee (Gemeinsamer Bundesausschuss)
