# Correction to: Implementation, efficacy, costs and processes of inpatient equivalent hometreatment in German mental health care (AKtiV): protocol of a mixed-method, participatory, quasi-experimental trial

**DOI:** 10.1186/s12888-021-03204-3

**Published:** 2021-04-19

**Authors:** Johanna Baumgardt, Julian Schwarz, Andreas Bechdolf, Konstantinos Nikolaidis, Martin Heinze, Johannes Hamann, Martin Holzke, Gerhard Längle, Janina Richter, Peter Brieger, Reinhold Kilian, Jürgen Timm, Constance Hirschmeier, Sebastian Von Peter, Stefan Weinmann

**Affiliations:** 1 Department of Psychiatry, Psychotherapy and Psychosomatic Medicine, Vivantes Hospital Am Urban und Vivantes Hospital im Friedrichshain, Charité – Universitätsmedizin Berlin, Vivantes Klinikum Am Urban, Berlin, Germany; 2 Department of Psychiatry and Psychotherapy, Brandenburg Medical School Theodor Fontane, Immanuel Clinic Rüdersdorf, Rüdersdorf, Germany; 3 ORYGEN, National Center of Excellence of Youth Mental Health, University of Melbourne, Melbourne, Australia; 4 Department for Psychiatry and Psychotherapy, University Hospital Cologne, Cologne, Germany; 5 kbo-Isar Amper Klinikum, Region München, Munich, Germany; 6 Department of Psychiatry and Psychotherapy, Technical University of Munich, Munich, Germany; 7 Center for Psychiatry Suedwuerttemberg, Department of Psychiatry I, Ulm University, Ravensburg, Weissenau Germany; 8 Center for Psychiatry Suedwuerttemberg, Zwiefalten, Germany; 9 Gemeinnützige GmbH für Psychiatrie Reutlingen (PP.rt), Academic Hospital of Tuebingen University, Reutlingen, Germany; 10 Department of Psychiatry and Psychotherapy, University Hospital Tuebingen, Department of Medicine of the Tuebingen University, Tuebingen, Germany; 11 Department of Psychiatry II, Ulm University, Günzburg, Germany; 12 University of Bremen, Bremen, Germany; 13 Department for Psychiatry and Psychotherapy, Charité University Hospital Berlin, Berlin, Germany; 14 Psychiatric Hospital and Rehabilitation Unit, Rudolf-Sophien-Stift, Stuttgart, Germany; 15 University Psychiatric Hospital Basel, Basel, Switzerland

**Correction to: BMC Psychiatry 21, 173 (2021)**

**https://doi.org/10.1186/s12888-021-03163-9**

Following the publication of the original article [1], the authors identified errors in the authors names. The given names and family names were erroneously transposed.

The incorrect authors names are:

Baumgardt Johanna1*†, Schwarz Julian2†, Bechdolf Andreas1,3,4, Nikolaidis Konstantinos1, Heinze Martin2, Hamann Johannes5,6, Holzke Martin7, Längle Gerhard8,9,10, Richter Janina10, Brieger Peter5, Kilian Reinhold11, Timm Jürgen12, Hirschmeier Constance13, Von Peter Sebastian2† and Weinmann Stefan14,15†

The correct authors names are:

Johanna Baumgardt1*†, Julian Schwarz2†, Andreas Bechdolf1,3,4, Konstantinos Nikolaidis1, Martin Heinze2, Johannes Hamann5,6, Martin Holzke7, Gerhard Längle8,9,10, Janina Richter10, Peter Brieger5, Reinhold Kilian11, Jürgen Timm12, Constance Hirschmeier13, Sebastian Von Peter2† and Stefan Weinmann14,15†

The author group has been updated above and the original article [1] has been corrected.
